# Work Stress, Health Status and Presenteeism in Relation to Task Performance Among Chinese Medical Staff During COVID-19 Pandemic

**DOI:** 10.3389/fpubh.2022.836113

**Published:** 2022-04-27

**Authors:** Huanhuan Jia, Panpan Shang, Shang Gao, Peng Cao, Jianxing Yu, Xihe Yu

**Affiliations:** School of Public Health, Jilin University, Changchun, China

**Keywords:** work stress, health status, presenteeism, task performance, medical staff

## Abstract

**Objectives:**

This study aims to evaluate the direct effects of work stress, health status and presenteeism on task performance, and further explore the mediating effects of health status and presenteeism, hoping to provide theoretical basis for improving the performance of medical staff.

**Methods:**

A cross-sectional study was conducted among medical staff in Jilin Province, Northeast China. The Challenge and Hindrance-Related Self-Reported Stress scale, Short Form-8 Health Survey scale, Stanford Presenteeism Scale and Task Performance Scale were adopted to assess the work stress, health status, presenteeism and task performance of medical staff.

**Results:**

A total of 4,347 questionnaires were distributed among medical staff, and 4261 were valid, for an effective rate of 98.02%. The mean scores for work stress, health status, presenteeism and task performance were 2.05 ± 0.84, 4.18 ± 0.68, 2.15 ± 0.79 and 4.49 ± 0.64, respectively. The ANOVA results showed that there were significant differences in the task performance scores between different genders, ages, marital statuses, professional titles, departments and work years (*P* < 0.05). Work stress (β = −0.136, *P* < 0.001) and presenteeism (β = −0.171, *P* < 0.001) were negative predictors of task performance. Health status (β = 0.10; *P* < 0.001) was positive predictor of task performance. Health status (β = −0.070; *P* < −0.001) and presenteeism (β = −0.064; *P* < 0.001) mediated the relationship between work stress and task performance (*P* < 0.001). Presenteeism mediated the relationship between health status and task performance (β = 0.07; *P* < 0.001).

**Conclusion:**

Work stress and presenteeism had significant negative impact on the task performance of medical staff; health status had a significant positive effect on task performance. Meanwhile, health status and presenteeism played a mediating role in the relationship between work stress and task performance, and presenteeism played a mediating role in the relationship between health status and task performance. Reasonable assignment of tasks can reduce the work stress, but to improve the performance of medical staff, we should pay more attention on improving health, such as making health-related safeguard measures, raising awareness, building a platform, etc.

## Introduction

Since the end of 2019, there have been outbreaks of infectious diseases caused by coronavirus disease 2019 (COVID-19) worldwide. COVID-19 was declared a pandemic by the World Health Organization in January 2020 and listed as a public health emergency and a matter of international concern (1, 2). Emerging and re-emerging pathogens are global challenges to public health (3). Medical staff, as the backbone of COVID-19 prevention and control, has been playing a leading role in controlling the epidemic through the unremitting struggle against the epidemic (4). According to a survey from Europe, the COVID-19 pandemic affects the work of employees on every continent (5). Numerous studies have found that the COVID-19 pandemic is placing increasing demands on health care workers, it caused the severe employment situation, long or irregular working hours and shifts, and excessive work stress which were putting the task performance of medical staff and its health, society, and economy in a dangerous environment (6–8). These problems have seriously affected the attention, perception and decision-making ability of medical staff, not only reducing enthusiasm and initiative but also lowering the overall quality of medical staff. These consequences hinder the struggle against the virus, and the development of society.

The evaluation of the performance of health professionals has been the focus of scientific research in recent decades, and the efficient work of health technicians was the basis for improving the quality of health services (9). There have been extensive studies of task performance around the world, and the research factors mainly included work stress, job satisfaction, conflict, attendance, leadership relationships, health and so on (10–13). Studies indicated that more stressors arose during pandemics, and that stress itself was associated with the development of various diseases (14, 15). At the same time, health problems have become more prominent during the epidemic. In addition, healthcare workers faced more severe challenges during the pandemic. The heavy workload forced them to stick to their posts, and their attendance significantly improved, so we speculated that presenteeism was also more serious. While saving the lives of others, medical staff were more likely to neglect their own health, and the presenteeism rate of medical staff was much higher than that of other professions. In the context of the pandemic and the growing demand for medical services, medical staff are facing increasing work pressure, with a series of physical and mental health problems, and the possibility of going to work sick is increasing. Therefore, in combination with literature and epidemiological background, we selected these variables related to performance: work stress, health status, and presenteeism, to explore their direct and indirect effects on performance in multipath. Task performance refers to the employee's output in terms of goals and responsibilities related to the job. To some extent, it can more directly reflect an employee's work ability and performance. Task performance is considered to be one of the key indicators of organizational performance, contributing to an organization's productivity, competitiveness and social and psychological work environment (9). Therefore, this study adopted task performance as an indicator to measure the performance of medical staff.

The following subsections describe the hypotheses considered in this study.

## Hypothesis Development

### Work Stress

Work stress refers to the mismatch between work requirements and individuals under the interaction of work situations and individual characteristics, affecting individuals' physiology, psychology and behavior (16). COVID-19 has disrupted everyone's daily lives, making it challenging to maintain boundaries between work and non-work, and research by Kumar et al. (14) pointed to the many stressors that emerge during a pandemic that can disrupt people's work and affect their performance. Research by Bhagat (17) showed that people are more likely to experience distress due to stressful life events, which can lead to disruptions in work and thus affect their task performance. Medical staff faces high occupational pressure caused by heavy workloads, extended working hours and so on. Such high occupational pressure seriously affects the physical and mental health of medical staff, leads to an increase in the probability of work error, and seriously affects work efficiency (18).

Based on the above discussion, the following hypothesis was proposed.

**Hypothesis 1 (H1):** Work stress has a significant, negative effect on task performance.

### Health Status

The World Health Organization defines health as a good state of physical, mental and social wellbeing (19). Health is the basic condition for the normal work and lives of professional people. Health problems not only people's own lives, but they also have a direct or indirect impact on the labor productivity of employers, increasing the economic burden of the workplace, namely the loss of productivity caused by health (20). Ford et al.'s study (21) showed a significant correlation between physical and mental health and task performance. Baldwin et al.'s study (22) showed that the health status of medical staff directly affects the quality of medical work. At the same time, researches have shown that negative emotions associated with work stress may worsen employees' physical and mental health, and that work stress was a key factor in health care workers' physical and mental health during the pandemic (23, 24).

Based on the above discussion, the following research hypotheses were proposed:

**Hypothesis 2 (H2)**: Health status has a significant positive effect on task performance.
**Hypothesis 3 (H3)**: Health status mediates the relationship between work stress and task performance.

### Presenteeism

Presenteeism refers to “the phenomenon of people who normally need rest and absence from work going to work despite poor health” (25). Studies have shown that presenteeism is important because of its negative impact on individual health and organizational productivity (26, 27). Health productivity loss includes sickness absence and presenteeism (28). Presenteeism leads to much higher productivity loss than sickness absenteeism and is the main mode of working inefficiency among professional people (29). Studies in the USA have shown that presenteeism results in more lost productivity than sick leave (30). Employees who are sick tend to make more mistakes and have lower levels of performance and productivity (31). The study also found that the incidence of presenteeism of medical staff was significantly higher than that of other professions: the presenteeism of medical staff was ~3–4 times higher than that of other occupational groups, and the occurrence of presenteeism of medical staff is common (25, 32). A literature review showed that health status is associated with presenteeism and lost productivity (27). Other studies have shown that work stress can lead to presenteeism (33). The Health and Safety Executive (2015) reported that stress-related illnesses accounted for 35% of all health-related illnesses and 43% of presenteeism in the UK (34).

Based on the above discussion, the following research hypotheses are proposed:

**Hypothesis 4 (H4):** Presenteeism has a significant negative effect on task performance.
**Hypothesis 5 (H5):** Presenteeism mediates the relationship between health status and task performance.
**Hypothesis 6 (H6):** Presenteeism mediates the relationship between work stress and task performance.

**Figure 1:**
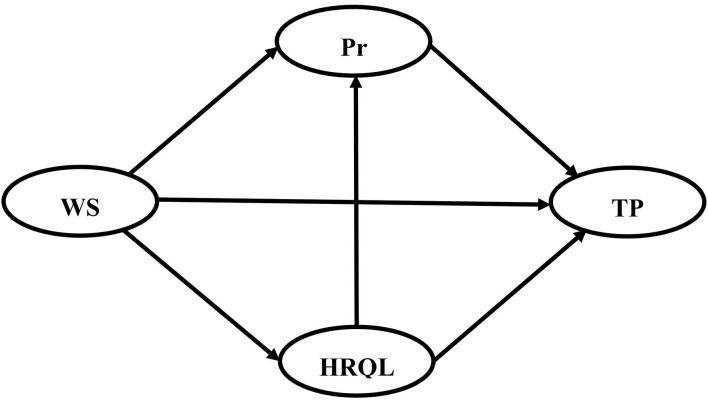
The hypothetical structural equation model. TP, task performance; WS, work stress; HRQL, health-related quality of life; Pr, presenteeism.

Based on the above discussion, a hypothetical path was proposed as shown in Figure 1.

## Methods

### Study Design and Participants

To validate the hypotheses, a cross-sectional survey was conducted in January 2020 in Jilin Province, Northeast China. Medical staff members in public hospitals were selected by stratified random sampling as the subjects of the study. In this process, all areas of Jilin Province were divided into cities and counties, and hospitals were also divided into urban public hospitals and county-level public hospitals. Due to the high clustering of urban public hospitals, these hospitals were stratified by region, type and level and randomly selected at a rate of 1/4. Because of the dispersion of different counties, city hospitals and traditional Chinese medicine hospitals were selected from each county as samples. Finally, a total of 109 hospitals, including 29 urban public hospitals and 80 county-level public hospitals, were selected as sample hospitals. Through quota sampling, 40 medical staff members, including doctors, nurses and medical technicians, were selected from each hospital to be investigated. The questionnaire was administered on site by trained investigators and was recovered on site. Finally, 4,347 questionnaires were distributed, and 4,261 valid questionnaires were collected. The valid response was 98.02%.

The study was approved by the Medical Ethics Committee of the author's institution (No. 2019-12-03). Participants were provided with a complete explanation of the purposes of the study. Participants were informed that the information collected would be used solely for the study and that they could withdraw at any time.

### Measures

The measuring instrument was a questionnaire, which was developed by adapting previously validated scales to the context of the study. The questionnaire was divided into two parts. The first part collected demographic characteristics of the respondents, including gender, age, marriage status, education background, professional title, department and working years. The second part measured respondents' task performance, work stress, health status and presenteeism.

### Challenge-and Hindrance-Related Self-Reported Stress Measures

Work stress was assessed using the Challenge-and Hindrance-Related Self-Reported Stress Measures (CHSS) developed by Cavanaugh et al. (35). It includes both challenging stress and hindrance stress. Challenge stressors were defined as work-related demands or circumstances that have associated potential gains for individuals despite potential stress. Hindrance stressors were defined as work-related demands or circumstances that tend to constrain or interfere with an individual's work achievements and that do not tend to be associated with the individual's potential gains. There are 11 items on the CHSS, including 6 challenging stressors and 5 hindrance stressors. The items are measured on a 5-point Likert scale ranging from 1 (no pressure) to 5 (extreme stress), and the higher that the score is, the greater that the work stress of the medical staff is. Cavanaugh's research (35) showed that the reliability coefficients of the two subscales were 0.87 and 0.75, and the correlation coefficient was 0.28, indicating that the two scales had good internal consistency and discriminant validity. The CHSS has been widely concerned and applied (36–41). In addition, Chinese scholars have translated it into Chinese and proved its applicability in Chinese professional groups (42). In this study, the Cronbach's alpha was 0.938 and 0.849 for the two subscales, respectively.

### Short Form-8 Health Survey

The Short Form-36 (SF-36) Health Survey is the most popular instrument for investigating health-related quality of life (HRQOL) (43), and it contains 36 items in 8 subscales. However, despite its popularity, the length of SF-36 limits its use (43). The SF-8, a shortened version of the SF-36, was derived from the SF-36 and has been preferred by many scholars (44–46). The SF-8 has only 8 questions, including physical functioning, role limitations due to physical health problems, bodily pain, general health perceptions, vitality, social functioning, role limitations due to emotional problems, and mental health; the scores range from 1 to 5, with higher scores indicating better performance. The SF-8 has demonstrated acceptable validity and reliability in previous studies (47). At the same time, the SF-8 was translated into Chinese by Lang et al. (43), and a survey of 10,885 individuals in 35 cities in China proved that the SF-8 had good internal consistency reliability and could be used in the Chinese population. The Cronbach's alpha for the SF-8 in this study was 0.916.

### Stanford Presenteeism Scale

The Stanford Presenteeism Scale (SPS-6) was adapted to measure the effect of health status on task performance (48). The SPS-6 is not affected by occupation, disease type or characteristics and has been used in many fields (27, 49). Based on a survey of 14,195 people in 8 places, Jiang proved that the SPS-6 has high reliability and validity and can be applied to the study of the Chinese professional population (50). There are 6 items in the SPS-6, each with five options ranging from 1 to 5. On the scale, “*At work, I was able to focus on achieving my goals despite my health problem” and* “*Despite having my health problem, I was able to finish hard tasks in my work*” are scored in reverse order. The sum of the six items then produces a total attendance score, and the higher that the score is, the greater that the health impact on work status is. In this study, the Cronbach's alpha for the SPS-6 was 0.756.

### Task Performance

The measurement of task performance (TP) was adapted from Williams's (51) and Jessica's (52) performance measurement scales. TP consists of five items, which are measured on a 5-point Likert scale, rated on a scale from 1 (Strongly disagree) to 5 (Strongly agree). The higher that the score is, the better that the employee's performance is. In this study, TP was translated into Chinese through translation and back-translation. Two PhD students each translated the original items and then decided on a draft after discussion. Then, the items were translated back into English to ensure that the Chinese version had the same meaning as the English version. In this study, the Cronbach's alpha for TP was 0.917.

### Data Analysis

Descriptive statistics were used to analyze the demographic characteristics of the respondents, as well as the scores for task performance, work stress, health status and presenteeism. In addition, age and working years were collected using continuous variables, whereas in order to reflect the task performance of different groups, we grouped them into groups with a distance of 10 based on previous studies (53, 54). The *t* tests and one-way analysis of variance (ANOVA) were performed to examine differences in the task performance scores among medical staff members of different genders, ages, marital statuses, educational levels, professional titles, departments, and working years. Pearson's correlation analysis was applied to analyze the correlations between the study variables. Structural equation modeling (SEM) has the characteristics of Confirmatory factor analysis (CFA) and Path analysis (PA), and has incomparable advantages. PA tests the causal relationship between the observed variables, CFA tests the causal relationship between the observed variables and the latent construct, and SEM tests the causality between the observed variables and the latent construal as well as the interior of several latent construal (55). Some scholars proposed that SEM was the sum of CFA, PA and multiple regression analysis (56). Therefore, SEM was adopted to verify the effects of work stress, health status and presenteeism on task performance, and the Maximum Likelihood Estimation was used to estimate the parameters. In addition, the bootstrapping technique was applied to explore the mediating role. The model was assessed by the following indexes (57, 58): (1) the standardized residual mean root (SRMR < 0.01); (2) the root mean square error of approximation (RMSEA ≤ 0.08); (3) the comparative fit index (CFI ≥ 0.90); (4) the Tucker–Lewis index (TLI ≥ 0.90); (5) the incremental fit index (IFI ≥ 0.90) and (6) the non-normed fit index (NFI ≥ 0.90). All of the statistical tests were two sided with the level of significance set at 0.05. SPSS software, version 25.0, and AMOS software, version 23.0 (IBM Corporation, Armonk, NY, USA), were used for the process.

## Results

### Demographic Characteristics and Task Performance Scores

A total of 4,261 medical staff participated in the survey. In this sample, most of the respondents were female (68.95%) and married (78.85%), with college degrees (58.60%) and with middle or junior titles (68.72%). The medical staff members were aged 37.55 ± 9.11 years old, had worked for 13.73 ± 9.73 years and scored 4.49 ± 0.64 on task performance. Mean scores for task performance differed across the distributions of gender, age, marital status, professional titles, departments and working years. However, there were no differences in the scores among medical staff members with different marital statuses and educational levels. The demographic characteristics and task performance scores are shown in Table 1.

**Table 1:** Demographic characteristics and task performance of 4,261 participants.

**Variables**	**N (%)**	**TP**	***P***
**Gender**			<0.001
Male	1,323 (31.05)	4.41 ± 0.70	
Female	2,938 (68.95)	4.52 ± 0.60	
**Age**			
≤ 30	1,167 (27.39)	4.45 ± 0.64	0.015
31–40	1,554 (36.47)	4.48 ± 0.62	
41–50	1,182 (27.74)	4.53 ± 0.64	
≥51	358 (8.40)	4.47 ± 0.69	
**Marital status**			
Unmarried	769 (18.05)	4.41 ± 0.65	0.003
Married	3,360 (78.85)	4.5 ± 0.63	
Divorced	97 (2.28)	4.55 ± 0.66	
Other	35 (0.83)	4.38 ± 0.86	
**Education**			
High school and below	268 (6.29)	4.52 ± 0.64	0.202
Junior college	1,145 (26.87)	4.48 ± 0.66	
College	2,497 (58.60)	4.5 ± 0.63	
Master's degree and above	351 (8.23)	4.43 ± 0.63	
**Professional title**			
Senior	178 (4.18)	4.53 ± 0.59	0.001
Sub-senior	798 (18.73)	4.53 ± 0.62	
Middle	1,303 (30.58)	4.5 ± 0.64	
Junior	1,625 (38.14)	4.47 ± 0.64	
None	357 (8.38)	4.37 ± 0.67	
**Department**			
Internal medicine	1,159 (27.2)	4.46 ± 0.63	0.005
Surgery	666 (15.63)	4.44 ± 0.68	
Gynecology	192 (4.51)	4.61 ± 0.49	
Pediatrics	144 (3.38)	4.46 ± 0.70	
Traditional Chinese medicine	146 (3.43)	4.4 ± 0.69	
Preventive medicine	21 (0.49)	4.61 ± 0.44	
Other	1,933 (45.36)	4.51 ± 0.63	
**Work years**			
≤ 5	1,277 (29.97)	4.41 ± 0.64	<0.001
6–15	1,525 (35.79)	4.5 ± 0.63	
16–25	863 (20.25)	4.52 ± 0.65	
≥26	596 (13.99)	4.56 ± 0.63	
Total	4,261 (100.00)		

*TP, task performance*.

### Correlations of Study Variables

Table 2 demonstrates the scores of the study variables. The health status score was 4.18 ± 0.68, and the presenteeism score was 2.15 ± 0.79. The work stress score was 2.05 ± 0.84, in which the challenging stress score was 2.22 ± 0.97 and the hindrance stress score was 1.85 ± 0.84. Health status was negatively correlated with work stress and presenteeism (*r* = −0.606, *P* < 0.01; *r* = −0.503, *P* < 0.01) and positively correlated with task performance (*r* = 0.279, *P* < 0.01). Work stress was positively correlated with presenteeism (*r* = 0.463, *P* < 0.01) and negatively correlated with task performance (*r* = −0.266, *P* < 0.01). Presenteeism was negatively correlated with task performance (*r* = −0.161, *P* < 0.01).

**Table 2:** Correlations among task performance, work stress, health-related quality of life and presenteeism.

**Variables**	**Mean**	**SD**	**HRQL**	**WS**	**Pr**	**TP**
HROL	4.18	0.68	1			
WS	2.05	0.84	−0.0606**	1		
Pr	2.15	0.79	−0.0503**	0.463**	1	
TP	4.49	0.64	0.279**	−0.266**	−0.161**	1

*TP, task performance; WS, work stress; HRQL, health-related quality of life; Pr, presenteeism*;

** *P < 0.01*.

### Hypothesis Testing

**Figure 2:**
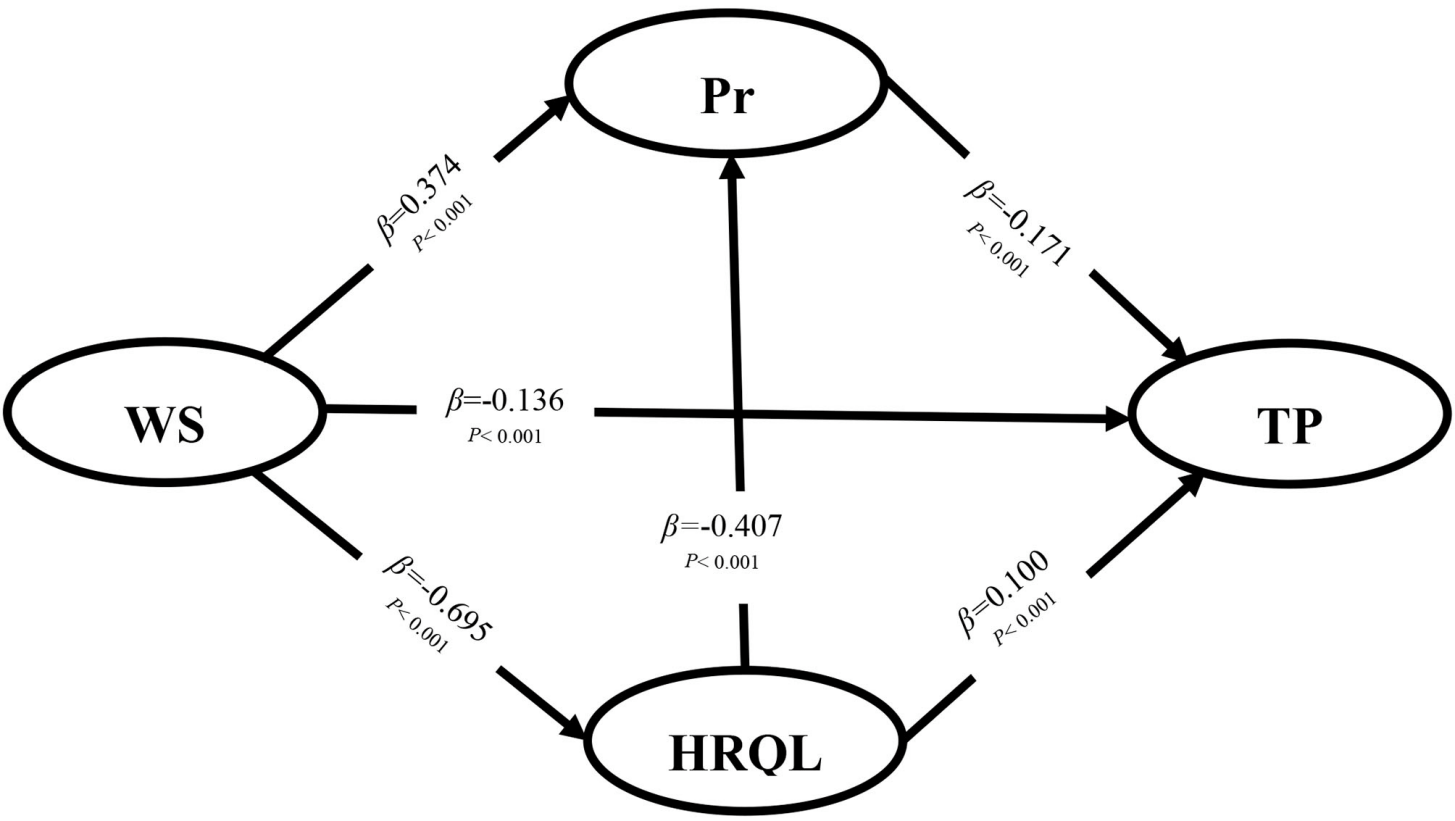
The results of hypothesis testing. TP, task performance; WS, work stress; HRQL, health-related quality of life; Pr, presenteeism.

The *t* and *P* values for each path were calculated in AMOS to test the research hypothesis. The results of the structural equation model are presented in Figure 2.

The results showed that the higher that the work stress is, the more serious that presenteeism is, and the worse that the task performance is (β = −0.136; *P* < 0.001; β = −0.171; *P* < 0.001); moreover, the better that health status is, the better that task performance is (β = 0.10; *P* < 0.001), so H1, H2, and H4 were supported. At the same time, the bootstrapping technique in AMOS was used to explore the mediating roles of presenteeism and health status, and the 95% confidence intervals of the indirect effects were obtained with 5,000 bootstrap resamples. The results showed that work stress significantly affected the task performance of medical staff through presenteeism (β = −0.064; *P* < 0.001) or health status (β = −0.070; *P* < 0.001), and health status significantly affected the task performance of medical staff through presenteeism (β = 0.070; *P* < 0.001), so H3, H5 and H6 were supported as shown in Table 3. In addition, the total effect of work stress and health status on task performance was −0.318 (*P* < 0.001) and 0.170 (*P* < 0.001), respectively. Table 4 shows the fit of the model, and the values represent an acceptably fitting model.

**Table 3:** Results of the effects on task performance.

**Path**	**Effect**	**Coefficient**	**Boot SE**	**Z**	***P***	**Bias-corrected**		**Percentile**	
						**95%CI**		**95%CI**	
						**Lower**	**Upper**	**Lower**	**Upper**
WS → TP	Direct	−0.136	0.031	−4.39	<0.001	−0.197	−0.075	−0.199	−0.076
	Indirect1	−0.070	0.020	−3.50	<0.001	−0.109	−0.029	−0.109	−0.030
	Indirect2	−0.064	0.011	−5.82	<0.001	−0.088	−0.044	−0.087	−0.043
	Total	−0.318	0.019	−16.74	<0.001	−0.354	−0.28	−0.355	−0.282
HRQL → TP	Direct	0.100	0.029	3.45	<0.001	0.042	0.156	0.043	0.156
	Indirect	0.070	0.012	5.83	<0.001	0.048	0.094	0.047	0.093
	Total	0.170	0.028	6.07	<0.001	0.114	0.223	0.114	0.223
Pr → TP	Direct	−0.171	0.027	−6.33	<0.001	−0.227	−0.119	−0.225	−0.118

*TP, task performance; WS, work stress; HRQL, health-related quality of life; Pr, presenteeism; Indirect1, WS → HRQL → TP; Indirect2, WS → HRQL → TP*.

**Table 4:** The fit of the structural equation model.

**Model**	**SRMR**	**RMSEA**	**CFI**	**TLI**	**IFI**	**NFI**
Reference	<0.1	<0.08	>0.9	>0.9	>0.9	>0.9
Model	0.038	0.046	0.975	0.970	0.975	0.972

## Discussion

This study explored the factors that influence the task performance of medical staff. A conceptual framework representing the direct and indirect relationships among four factors (task performance, work stress, health status, and presenteeism) was established and validated, and it was helpful for understanding the influence of physical and mental aspects on task performance. The data collection and processing in this study were strictly controlled, and the results could be considered a valuable reference for improving the task performance of medical staff. Based on the results of this study, it is now possible to revisit and reflect on the hypotheses established at the beginning of this study.

The survey results of this study showed that the mean score of task performance was 4.49, while the mean score of task performance of nurses in Malaysia was 3.85 as shown by Nasurdin et al. (59). This difference may be due to the fact that the research group includes different medical staff groups such as doctors and nurses, and nurses face more complicated work tasks, heavier work burden and greater occupational pressure than doctors (60). While reducing the burden of nurses, we should also strengthen the training of their comprehensive ability. The task performance score was relatively high, which may be due to the high sense of responsibility of medical staff in the context of the pandemic, which improves their work efficiency. It is very important to strengthen the guidance of medical ethics for medical staff. The mean score of challenging stress was 2.22, and that of obstructive stress was 1.85. Challenging stress includes workload, time pressure, job responsibilities, etc. Obstructive stress includes ambiguous roles, organizational politics, job insecurity, and blocked career development. This study found that the high score of challenging pressure indicates that the pressure of medical staff mainly comes from time pressure and workload, and also reflects the professional particularity of medical staff, which is more urgent than other industries. Therefore, hospitals and departments should make scientific and reasonable work arrangements to reduce the burden of medical staff. The average score of health status was 4.18, which was at a high level, indicating that the health status of medical staff in the investigated area was good, which may be affected by the local working and living environment and medical staff's own health management awareness. The average score for presenteeism was 2.05. In a study of nurses in Spain, Portugal and Brazil, the overall score for presenteeism was 3.36 (61). The reason for this difference may be, on the one hand, the medical staff group including doctors, nurses, medical skills, etc., whose tasks are not the same, on the other hand, it may be related to the better health status of medical staff in the surveyed areas. Local medical staff should continue to maintain good living and working habits and further strengthen health management.

Studies have shown that work stress, health status, and presenteeism were associated with task performance. Work stress and presenteeism had significant negative influence on medical staff's task performance, and health status had significant positive influence on it. The hypotheses proposed in this paper have been well verified in the structural equation model. The occupation of medical staff is special. Medical work is a high-risk occupation. The work of medical staff is characterized by high risk and urgency, especially during the epidemic period, facing with huge risks and long workloads, competing with time for patients' lives, research has shown that extreme work stress was a great challenge to the health status of medical staff, on the contrary, it would lead to anxiety, tension and other negative emotions, serious and even health problems, not conducive to work (62). Health problems, on the one hand, will increase long-term sick leave, on the other hand, medical staff is more likely to ignore its own health problems, resulting in higher presenteeism. Theoretically attendance will have a positive impact on performance, but sick attendance has a negative impact on performance. Sick attendance was strongly associated with burnout, and attendance due to presenteeism was considered to be working but poor performance, consistent with other study (63). As a result, medical institutions and relevant departments need to appropriately relieve the stress on medical staff through measures such as rational assignment of tasks and upgrading of professional skills, while paying close attention to the health of medical staff, through carrying out health lectures, regular medical check-up and other ways to improve their health literacy, reduce the possibility of carrying diseases to work, prevention or even avoidance of medical staff due to work stress, health status, presenteeism of work efficiency.

The study also found that health status and presenteeism played an mediating role between work stress and task performance, and presenteeism played an mediating role between health status and task performance. The hypotheses were verified in the structural equation model. Research has shown that work stress was linked to health status, when working in a high-pressure environment, health problems were more likely to occur, health was threatened, medical staff also insisted on working with diseases, not only would lead to work errors, work inefficiency, it also exacerbated the negative health effects of the disease, creating a vicious circle and even placing a physical or work burden on others, which was detrimental to the health and well-being of individuals and society as a whole (64, 65). Occupational characteristics lead to stress in the medical profession is inevitable, blindly reducing the work stress of medical staff may reduce the quality of service of medical staff. So when work stress is a factor that can be hard to control, we can focus on health and presenteeism. As hospital administrators, they should take effective measures, such as providing health-promoting places, adding more fitness facilities, enhancing their physical quality, carrying out psychological intervention and relevant training, and enhancing their awareness of health self-management, improve their health and productivity. The factors involved in this study were limited. Future researches may further explore other factors related to performance, and suggest that we should continue to pay attention to the health status of medical staff.

Regarding advantages of this study, previous studies have not addressed the relationships among work stress, health status, presenteeism, and task performance. We established these associations and explored the direct and indirect roles among them. This study investigated a large population, carried out strict quality control, and was highly scientific and representative. Regarding disadvantages, although this study explored and verified the relationships among the four variables, it inevitably has limitations. First, this study was a cross-sectional study, so it is impossible to determine the causal relationships among the variables. In the future, longitudinal study design should be used to collect data as far as possible. Second, this is only one province of China, there may be differences between different provinces, which might have affected the generalizability of the results. When conditions permit, representative sample data can be selected from the whole country for analysis. Third, the method of quota sampling may produce research bias. However, after consulting with local health authorities and health experts, stratified random sampling was applied to capture hospitals and medical staff to minimize bias.

## Conclusion

In conclusion, our results showed that work stress and presenteeism had a significant negative effect on task performance of medical staff; health status had a significant positive effect on task performance. At the same time, health status or presenteeism played a mediating role between medical staff's work stress and task performance, and presenteeism played a mediating role between medical staff's health status and task performance. To improve task performance, priority should be given to starting from ideological cognition, platform construction, safety assurance and other aspects, and continuously strengthen the health management of medical institutions and medical personnel.

## Data Availability Statement

The raw data supporting the conclusions of this article will be made available by the authors, without undue reservation.

## Ethics Statement

The studies involving human participants were reviewed and approved by Medical Ethics Committee of the School of Public Health Jilin University. The patients/participants provided their written informed consent to participate in this study.

## Author Contributions

HJ and PS: methodology and writing—original draft preparation. SG, JY, and PC: data curation and investigation. XY: funding acquisition, conceptualization, and supervision. All authors contributed to the article and approved the submitted version.

## Funding

This work was supported by the MOE (Ministry of Education of the People's Republic of China) Project of Humanities and Social Sciences (Grant Number 18YJAZH118). The funding agencies had no role in design, analysis, interpretation, or writing of this study.

## Conflict of Interest

The authors declare that the research was conducted in the absence of any commercial or financial relationships that could be construed as a potential conflict of interest.

## Publisher's Note

All claims expressed in this article are solely those of the authors and do not necessarily represent those of their affiliated organizations, or those of the publisher, the editors and the reviewers. Any product that may be evaluated in this article, or claim that may be made by its manufacturer, is not guaranteed or endorsed by the publisher.
